# Deciphering the effects of aging on Alzheimer’s disease based on mitochondrial dysfunction and neuroinflammation

**DOI:** 10.3389/fnagi.2026.1991837

**Published:** 2026-10-27

**Authors:** Yamei Luo, Haoli Yu, Jun Zhang, Zhihong Wu, Haiyan Zhang

**Affiliations:** 1 School of Medical Technology, Chongqing Three Gorges Medical College, Chongqing, China; 2 School of Public Health and Management, Chongqing Three Gorges Medical College, Chongqing, China; 3 School of Traditional Chinese Medicine, Chongqing Three Gorges Medical College, Chongqing, China

**Keywords:** aging, Alzheimer’s disease, mitochondrial dysfunction, mitochondrial quality control, mtDAMPs, neuroinflammation

## Abstract

Alzheimer’s disease (AD), a neurological disorder characterized by progressive deterioration of cognitive function, has complex pathogenic mechanisms and lacks curative therapies with proven efficacy. Aging and inflammatory responses, which play indispensable roles in the onset and progression of AD, are closely intertwined with mitochondrial dysfunction. During aging, a series of degenerative alterations, including accumulated mitochondrial DNA (mtDNA) mutations, excessive mitochondrial reactive oxygen species (ROS) accumulation, disrupted mitochondrial quality control (MQC) and disturbed energy metabolism, further induce structural mitochondrial damage. This damage causes the leakage of damage-associated molecular patterns (DAMPs) and activates numerous downstream inflammatory signaling pathways. In brain tissue, inflammatory cytokines, which continuously facilitate blood–brain barrier (BBB) disruption, aberrant activation of microglial cells, tau protein hyperphosphorylation, as well as Aβ accumulation, drive an endlessly amplified neuroinflammatory cascade that leads to irreversible neuronal damage and fuels the initiation and progression of AD. In this review, we clarify the regulatory effects of aging on AD pathogenesis through the mitochondrial dysfunction-chronic inflammation axis and ultimately focus on mitochondrial-targeted therapies, with future outlooks for AD treatment provided.

## 1 Introduction

Alzheimer’s disease (AD) is an age-related neurodegenerative disorder whose cardinal manifestations encompass cognitive impairment, inflammatory responses and mitochondrial dysfunction. The growing elderly cohort will trigger a rapid rise in late-onset Alzheimer’s disease (LOAD, onset at age ≥65), whose worldwide number is estimated to double by 2050 (Scheltens et al., 2021), imposing severe economic and healthcare burdens on societies globally. It is noteworthy that statistics reveal the prevalence of AD stands at approximately 10% among people over 65 years old, while this figure climbs to 32% for individuals aged above 85, which suggests that aging exerts a vital impact on the progression of AD pathology (Leng and Edison, 2021).

The pathogenesis of AD is thought to correlate with amyloid-β (Aβ) deposition within neurons and neurofibrillary tangles generated by tau hyperphosphorylation (Pan et al., 2026). In fact, the amyloid cascade hypothesis, taken in isolation, cannot offer a comprehensive account of how AD originates and advances over time. First, Aβ deposits can be detected in approximately 30% of cognitively normal elderly individuals. Second, therapeutic strategies designed to clear cerebral Aβ have yielded limited clinical efficacy to date (Wang et al., 2023). Meanwhile, a growing number of studies have highlighted the vital contribution of neuroinflammation to AD progression. Recent studies find that microglia exhibit acute inflammatory transcriptional profiles in the early stage of AD, whereas chronic inflammatory activation predominates in the late phase (Sun et al., 2023). In addition, phosphoenolpyruvate, a glycolytic metabolite, can exert therapeutic effects in AD through the inhibition of inflammatory signals (Song et al., 2026).

Taken together, both aging and neuroinflammation exert profound effects on the initiation and pathological advancement of AD. Wilkins et al. further argue that mitochondria serve as the central hub linking aging and AD (Wilkins and Swerdlow, 2021), while accumulating evidence corroborates links between AD etiology, mitochondrial dysfunction, and chronic inflammation (Guo et al., 2025; Gertie and Chung, 2025; Song et al., 2025). As one of the hallmark characteristics of aging, mitochondrial dysfunction can simultaneously couple the activation of inflammatory pathways with AD pathological lesions. Previous reviews have independently summarized the relationships between mitochondrial dysfunction and AD (D'Alessandro et al., 2025; Klemmensen et al., 2024; Wang et al., 2024), or neuroinflammation and AD (Heneka et al., 2025; Wang et al., 2023; Chen and Yu, 2023). Those reviews focus on either the upstream mitochondrial defects or the downstream neuroinflammatory responses, yet rarely systematically integrate the causal chain and bidirectional crosstalk among aging, mitochondrial damage, chronic neuroinflammation, and AD pathological progression. In contrast, the present review constructs an “aging-mitochondrial dysfunction-chronic inflammation-AD” axis, emphasizing that aging acts as the initial driving factor, mitochondrial dysfunction serves as the core intermediate pathological link, and persistent chronic inflammation further amplifies neuronal damage and tau/Aβ pathology. We hypothesize that aging-triggered mitochondrial damage and chronic inflammation may serve as early initiating drivers of AD, rather than merely secondary consequences following Aβ and tau pathology. Accordingly, we focus on the upstream and downstream regulatory mechanisms of inflammation to elaborate the specific effects of aging on AD, and further explore therapeutic strategies for AD from a mitochondrial perspective (Figure 1).

**Figure 1:**
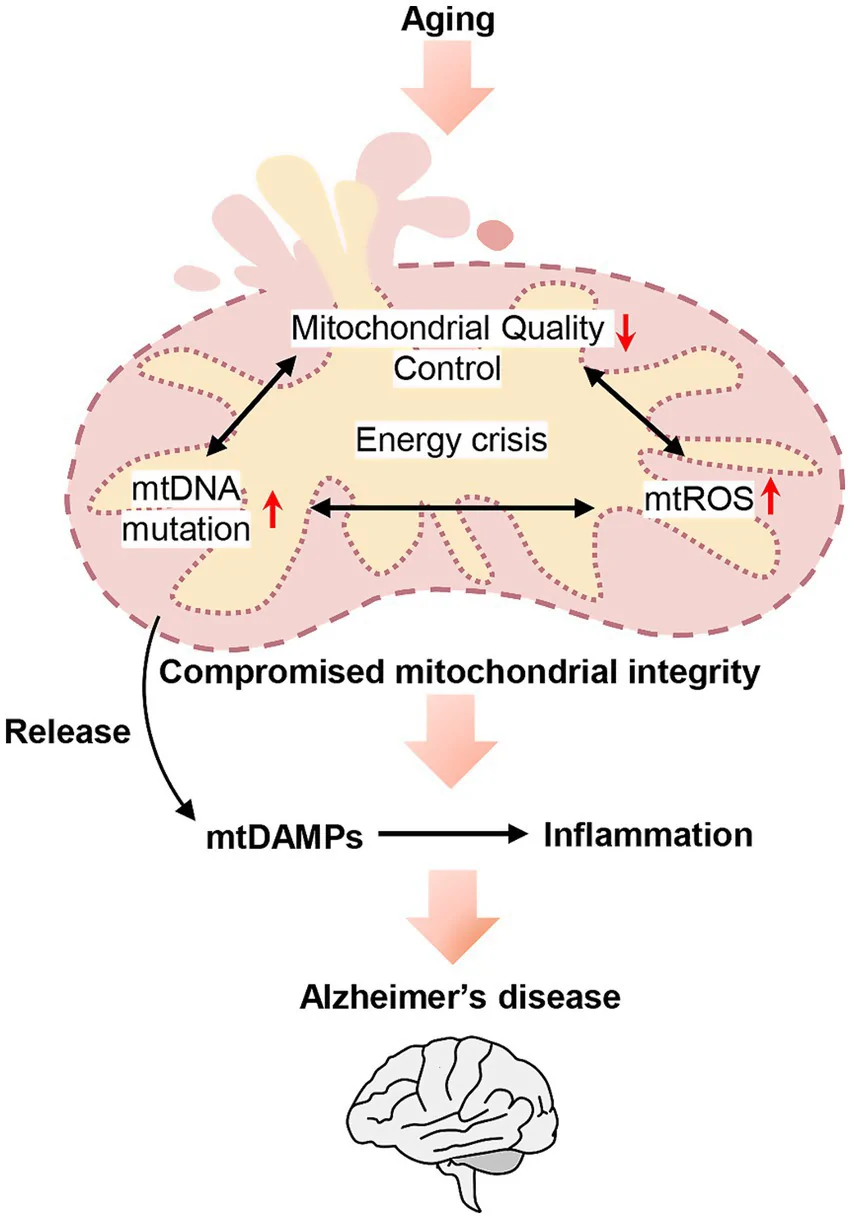
Aging promotes Alzheimer’s disease via the mitochondrial dysfunction-inflammation axis. Upward arrows indicate increase; downward arrows represent functional impairment. mtDNA, mitochondrial DNA; mtROS, mitochondrial reactive oxygen species; mtDAMPs, mitochondrial-derived damage-associated molecular patterns.

## 2 Mitochondrial dysfunction in aging

The endosymbiotic theory posits that mitochondria are semi-autonomous organelles evolved gradually from ancient aerobic α-proteobacteria engulfed by ancestral eukaryotic cells. Notably, mitochondria undergo irreversible alterations during the aging process. Statistical analyses based on populations of different ages and genders have demonstrated that the abundance of mitochondrial DNA (mtDNA) and mitochondrial RNA (mtRNA), as well as ATP production, decline significantly in elderly individuals, indicating progressive deterioration of mitochondrial function with advancing age (Short et al., 2005). To mitigate systemic defects arising from mitochondrial dysfunction, cells exert a compensatory inhibitory effect on mitophagy to elevate the total mitochondrial mass. Nevertheless, this adaptive response results in the buildup of impaired mitochondria, establishing a vicious cycle of aging-related mitochondrial homeostatic imbalance (Correia-Melo et al., 2016; Tran and Reddy, 2021; Palikaras et al., 2015). Multiple age-related diseases are closely linked to mitochondrial dysfunction, such as cardiovascular diseases (Liu c. et al., 2022), cancers (Lu et al., 2009), and neurodegenerative disorders (Wallace, 2008). Therefore, elucidating the specific alterations in mitochondria triggered by aging and subsequent downstream responses is of great significance for the treatment of age-related disorders.

### 2.1 Accumulated mtDNA mutations

High copy number, active asymmetric replication (Yasukawa and Kang, 2018), absent histone shielding and incomplete repair machinery (Anderson et al., 2020; Macao et al., 2015) render mtDNA prone to mutations [the mtDNA mutation rate is more than 15 times that of nuclear DNA (nDNA) (Frison et al., 2025)]. According to statistics, the mutation forms of mtDNA are mainly point mutations, insertions, and deletions, among which point mutations are the most common (Szczepanowska and Trifunovic, 2017; Kauppila et al., 2018). Aging causes the accumulation of mtDNA mutations in somatic cells (Huang et al., 2025). Recent studies have shown that single-nucleotide mutations in human blood mtDNA generated by replication rise sharply after age 60 (Gupta et al., 2026). mtDNA mutations are closely related to age-related declines, such as intestinal aging (Yang L. et al., 2024) and reproductive aging in females (Yang et al., 2020). In addition to sequence changes, the dysregulation of m6A methylation modification of mtDNA also shortens lifespan in nematodes (Hahn et al., 2024). All these findings demonstrate that mtDNA mutations exert profound impacts on the aging process.

### 2.2 Elevated mitochondrial reactive oxygen species (mtROS)

As early as 1956, Denham Harman’s free radical theory already highlighted the driving effect of ROS on aging (Harman, 1956). In fact, 90% of intracellular ROS are generated in mitochondria (Bhargava and Schnellmann, 2017). Studies have shown that, due to increased ROS production and weakened clearance capacity, mitochondrial function will undergo progressive decline (Harrington et al., 2023). In progeria model mice, it has been further found that the aging phenotype of their muscle fibers is associated with increased oxidative damage to mtDNA (Li Y. et al., 2024). Numerous studies have confirmed that reducing oxidative stress levels can effectively extend lifespan (Harrington and Harley, 1988; Shibamura et al., 2009), and long-lived populations generally exhibit lower levels of ROS and oxidative damage (Gruber et al., 2008; Gredilla et al., 2001). More importantly, senescent cells universally show a marked elevation in mtROS (Lai et al., 2025).

ROS are highly-reactive species that can directly react with DNA, proteins and lipids, thereby inflicting damage to biological macromolecules (Weidinger and Kozlov, 2015). The oxidative modification of nuclear genes can induce heritable mutations, such as the oxidation of guanine (G) to 8-oxoguanine (8-OG) (Michaels et al., 1992; Tsuzuki et al., 2001), which is significantly elevated in elderly populations (Choudhury et al., 2022). Beyond DNA, oxidative modification of proteins also intensifies with aging (Stadtman, 1992; Xu F. et al., 2025). More critically, the accumulation of this oxidative damage drives the development of age-related diseases, including cardiovascular diseases, neurodegenerative disorders, and diabetes (Fra et al., 2017; Baraibar et al., 2012b; Xu F. et al., 2025). Interestingly, mitohormesis posits that low levels of ROS are beneficial for health and longevity, and that pre-exposure to ROS stimuli during development promotes the acquisition of defensive capacity that is retained into old age, thereby enabling the organism to resist age-related decline (Cheng et al., 2023). Although the two views appear to hold opposing positions on whether ROS are beneficial or harmful, they actually both agree that once ROS levels exceed the physiological tolerance range, they accelerate the aging process.

### 2.3 Imbalanced mitochondrial quality control (MQC)

Tsui et al. categorized MQC into four core modules, including renewal, removal, repair, and remodeling (Tsui et al., 2026). Aging causes synergistic imbalance among the four parts, which further results in the accumulation of dysfunctional mitochondria and eventually induces senescence (Tsui et al., 2026).

The peroxisome proliferator-activated receptor-γ co-activator 1α (PGC-1α) serves as a key factor governing mitochondrial biogenesis. The expression of PGC1α4 in human skeletal muscle is negatively correlated with age, which contributes to age-related muscle atrophy (Guo et al., 2023). Similar findings were observed in the brain of aged mice, accompanied by upregulated inflammatory signaling and diminished activity of pathways related to neuronal function (Souder et al., 2025). Importantly, although total mitochondrial content remains unchanged with aging, the reduced ATP production and PGC-1α expression point to impaired mitochondrial turnover (Preston et al., 2008). Numerous studies have confirmed that enhancing mitochondrial biogenesis serves as a critical strategy for delaying aging. For instance, overexpression of *dPGC-1* in *Drosophila melanogaster* prolongs lifespan by boosting mitochondrial biogenesis (Rera et al., 2011). Plasma extracellular vesicles from young mice can rescue mitochondrial dysfunction in old mice by upregulating PGC-1α (Chen et al., 2024).

Besides reduced mitochondrial renewal, impaired clearance of damaged mitochondria is another major reason for MQC imbalance. The elimination of defective mitochondria mainly relies on mitophagy, the mitochondrial proteasome pathways, mitochondrial-derived vesicles (MDVs), as well as the mitochondrial unfolded protein response (UPR^mt^) (Ng et al., 2021). Since UPR^mt^ primarily exerts its physiological roles in development (Shpilka and Haynes, 2018), we do not discuss this pathway here.

Mitophagy undergoes age-dependent deterioration (Palikaras et al., 2015), yet conflicting findings have been reported in other research. Structural mitochondrial lesions and inflammatory responses triggered by aging can activate PINK1/Parkin-mediated mitophagy (Jimenez-Loygorri et al., 2024). Nevertheless, this compensatory upregulation cannot completely curb the accumulation of damaged mitochondria, which precisely mirrors the imbalance of MQC during aging (Jimenez-Loygorri et al., 2024). Furthermore, modulation of mitophagy can regulate the aging process. Inhibition of mitophagy can directly induce cellular senescence (Kelly et al., 2024). Meanwhile, activation of BNIP-3-dependent mitophagy in *Drosophila* neurons effectively delays systemic aging (Schmid et al., 2022). Urolithin A, an autophagy activator, eliminates dysfunctional mitochondria and mitigates chronic inflammation, leading to improvements in cognitive performance and retinal function (Jimenez-Loygorri et al., 2024). In addition, Urolithin A can extend the lifespan of *Caenorhabditis elegans* and ameliorate muscle function in mice (Ryu et al., 2016).

Mitochondrial Lon peptidase 1 (LONP1), an ATP-dependent protease, is responsible for the selective degradation of aberrant and oxidatively damaged proteins (Shetty et al., 2024). Notably, hepatic LONP enzymatic activity was significantly decreased in aged rats, while the abundance of oxidized LONP was substantially elevated (Bakala et al., 2003). Besides, age-mediated reduction in LONP1 expression was detected in human and rat osteoarthritic cartilage, and such downregulation seems to correlate with osteoarthritis progression (He Y. et al., 2022).

MDVs represent a pathway that transports specific damaged mitochondrial components to peroxisomes, lysosomes, or the extracellular space for degradation (Konig and McBride, 2024). In the early stage of aging, oxidized SOD1 facilitates MDV formation as a defensive response to damage, whereas the capacity of mitochondria to generate MDVs becomes exhausted in advanced aging (Guo Y. et al., 2024). This suggests that MDV functionality is dynamically modulated during aging.

Lysosomes, acting as a crucial step of MQC, establish physical tethering with mitochondria to eliminate defective mitochondrial components (Kleele et al., 2021). However, during aging, the lysosomal membrane structure is disrupted, and both acidity and degradative capacity decline (Steinhauser et al., 2026; Sun et al., 2020). Interestingly, recent studies have verified that mitochondria, rather than the cytosol, serve as the primary proton donors for lysosomes (Liu et al., 2026; Tian et al., 2026). Aging reduces mitochondrial-lysosomal contacts, which subsequently leads to lysosomal alkalinization, suppressed autophagic function, and excessive inflammatory response (Liu et al., 2026, Tian et al., 2026).

Taken together, reduced mitochondrial biogenesis coupled with impaired degradation mechanisms leads to irreversible mitochondrial decay, serving as a pivotal upstream trigger for inflammation.

### 2.4 Inefficient energy production

With advancing age, accumulated damage and dysregulated homeostasis collectively severely compromise normal mitochondrial function, manifested as reduced respiration linked to ATP synthesis (Samokhvalov et al., 2004). Age-related alterations in substrate metabolism and oxidative phosphorylation serve as critical limiting factors for energy generation.

Oxidative metabolic disorders triggered by aging specifically present with suppressed fatty acid oxidation and impaired oxidative phosphorylation (Elmansi and Miller, 2024). Statistical data indicate that among genes encoding mitochondrial proteins with significantly altered expression during aging, approximately 95% are downregulated, and the majority of these genes are involved in substrate metabolism, oxidative phosphorylation, and the tricarboxylic acid (TCA) cycle (Preston et al., 2008). Furthermore, the enzymatic activities of electron transport chain (ETC) complexes are decreased during aging (Preston et al., 2008; Elmansi and Miller, 2024; Migliavacca et al., 2019). Notably, the activity of F₀F₁ ATP synthase, the key enzyme responsible for ATP synthesis, also decreases significantly with aging (Guerrieri et al., 1992; Saqib et al., 2026). Interestingly, recent studies have found that aging induces altered subunit stoichiometry (reduced SUe/SUc ratio) destabilizes ATP synthase oligomerization, increases its mobility and redistributes the complex, which induces enlarged cristae fenestrations and diminishes mitochondrial ATP production in cardiomyocytes (Morris et al., 2026).

Aging leads to reduced lipid oxidation capacity in older adults, manifesting as increased body fat mass, and switches cellular oxidative fuel preference from fat to glucose (Elmansi and Miller, 2024). Aging also impairs mitochondrial calcium uptake, which further suppresses the activation of pyruvate dehydrogenase, thereby inhibiting the TCA cycle (Gherardi et al., 2025). Intriguingly, studies have identified that the transcriptional upregulation of mitochondria-related genes serves as a shared signature for most longevity-extending pathways (Tyshkovskiy et al., 2019), which implies that restoring mitochondrial function is a vital approach to delay aging.

## 3 Mitochondrial damage-associated molecular patterns (mtDAMPs) activate inflammatory responses

Various age-related mitochondrial dysfunctions induce mitochondrial permeability transition, which greatly facilitates the release of intramitochondrial contents (Ju et al., 2025; Green et al., 2011). Furthermore, Green et al. have demonstrated that the accumulation of damaged mitochondria promotes the onset of inflammation and neurodegenerative diseases (Green et al., 2011). Chronic inflammation associated with aging, namely inflammaging, is predominantly driven by the sustained activation of endogenous DAMPs (Xu X. et al., 2025). The antigenic nature of mitochondria, together with aging-induced collapse of mitochondrial membrane architecture, lays the groundwork for the release of DAMPs. Recent studies have confirmed that aging impairs inner mitochondrial membrane connectivity and widens membrane pores, accompanied by marked reductions in cristae density and width (Molina-Riquelme et al., 2026). Such ultrastructural remodeling results from an age-related decline in OPA1 expression (Molina-Riquelme et al., 2026) (Figure 2A). The disruption of cristae architecture exacerbates inflammatory responses and thereby accelerates premature aging (He B. et al., 2022).

**Figure 2:**
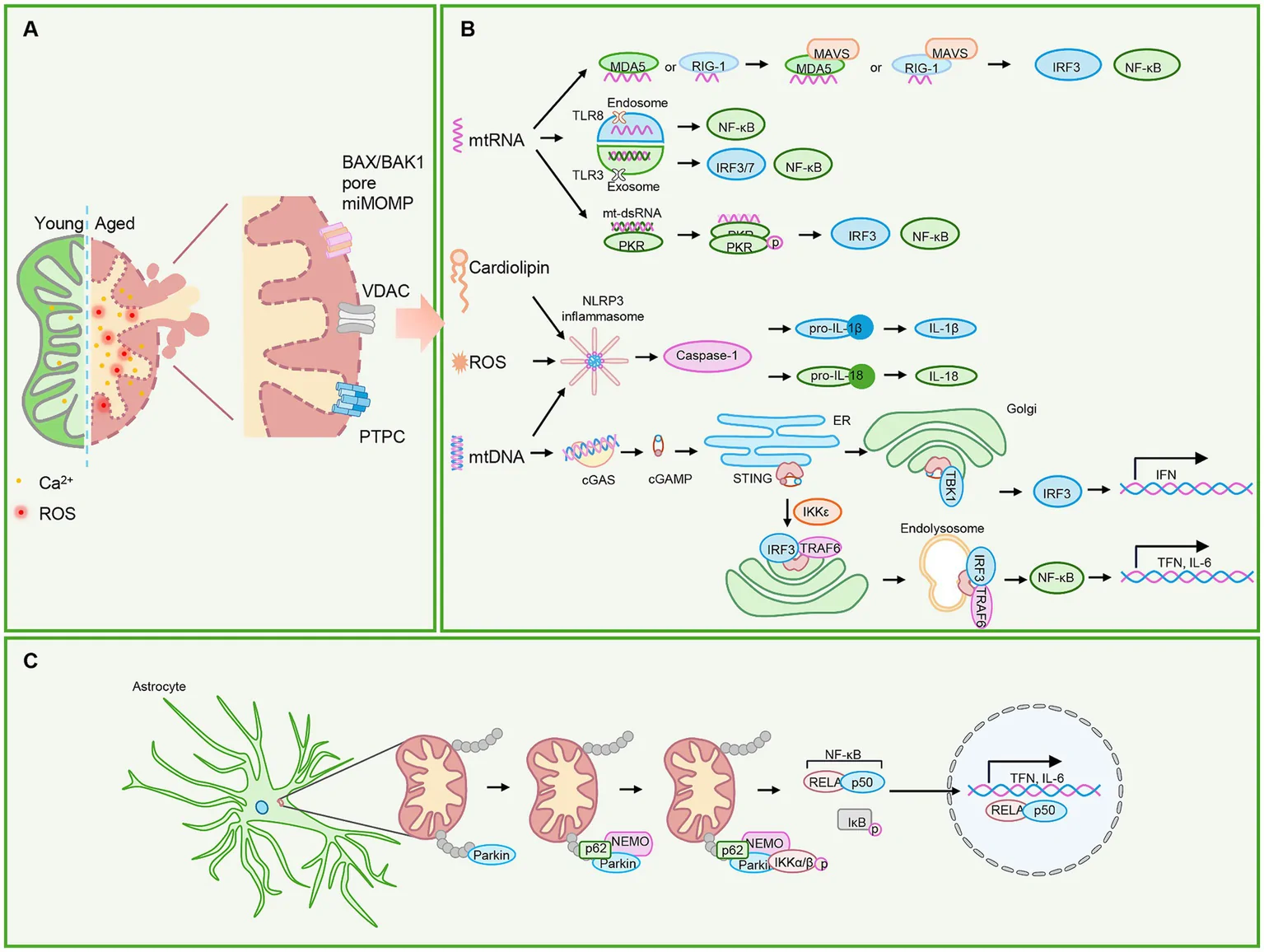
Schematic illustration of inflammation triggered by mtDAMPs. **(A)** Senescence triggers collapse of the mitochondrial membrane system. Aging reduces the density of mitochondrial inner membrane cristae and increases membrane permeability. **(B)** Core mechanisms underlying inflammatory signalling-pathway activation by mtDAMPs. Mitochondrial damage releases multiple mtDAMPs including mtDNA, mtRNA, cardiolipin and ROS. These molecules engage distinct innate immune sensors, triggering the activation of cGAS-STING, NLRP3 inflammasome, NF-κB and IRF3 signalling, which together drive the induction of pro-inflammatory gene expression. **(C)** Mitochondrial ubiquitination triggers inflammation instead of degradation. NEMO, together with catalytic subunits IKKα and IKKβ, forms the IKK complex. ROS, Reactive oxygen species; miMOMP, Minority mitochondrial outer membrane permeabilization; VDAC, Voltage-dependent anion channels; PTPC, Permeability transition pore complex; mtRNA, Mitochondrial RNA; RIG-I, Retinoic acid-inducible gene I protein; MDA5, Melanoma differentiation-associated protein 5; LGP2, Laboratory of genetics and physiology 2; MAVS, Mitochondrial antiviral signaling protein; IRF3, Interferon regulatory factor 3; TLR8, Toll-like receptor 8; TLR3, Toll-like receptor 3; mt-dsRNA, Mitochondrial double-stranded RNA; PKR, Protein kinase R; cGAS, Cyclic GMP-AMP synthase; cGAMP, Cyclic GMP-AMP; STING, Stimulator of interferon genes; ER, Endoplasmic reticulum; TBK1, Tank-binding kinase 1; IFN, Interferon; TRAF6, TNF-receptor-associated factor 6; NEMO, NF-κB essential modulator, also known as IKKγ; IKKα/β, IκB kinase α/β; RELA, Nuclear factor-κB p65 subunit.

Typically, mtDAMPs, including mtDNA, mtRNA, ROS, cardiolipin and so on, escape into the cytosol, functioning as danger ligands to bind to pattern recognition receptors, activate inflammation (Marchi et al., 2023) (Figure 2B). Li et al. (2023) reported elevated expression of multiple senescence-associated secretory phenotype (SASP) factors in senescent cells, especially pro-inflammatory cytokines such as IL-1α, IL-1β, and TNF-α, serving as key drivers of inflammatory responses. Overexpression of mitochondria-targeted 8-oxoG glycosylase 1 (OGG1) in mice alleviates neuroinflammation by reducing cytosolic mtDNA, suggesting that oxidative damage to mtDNA is a critical trigger for its leakage (Hussain et al., 2023). These findings illustrate that the accumulation of mitochondrial damage is a critical driver of increasing mitochondrial permeability, which then sets the stage for mtDAMPs release and subsequent activation of downstream inflammatory responses.

### 3.1 Mechanisms of mtDAMPs release

Multiple mechanisms responsible for mitochondrial content leakage have been identified to date, including pores formed by BAX/BAK1 on the outer mitochondrial membrane (OMM) (McArthur et al., 2018), voltage-dependent anion channels (VDAC) (Kim et al., 2019), as well as inner membrane-associated mitochondrial permeability transition (MPT) (Newman and Shadel, 2023). A small fraction of mitochondria in senescent cells undergo BAX/BAK1-dependent mitochondrial outer membrane permeabilization (MOMP) without driving cell death, a phenomenon defined as minority MOMP (miMOMP) (Ichim et al., 2015), which activates the cyclic GMP-AMP synthase (cGAS)-stimulator of interferon genes (STING) signaling pathway through the release of mtDNA during the aging process (Victorelli et al., 2023). More importantly, MOMP inhibition has been shown to extend the healthspan of aged mice (Victorelli et al., 2023).

Besides, pathways governing the release of oxidatively modified mtDNA involve mitochondrial permeability transition pore (mPTP) and VDAC-dependent channels (Xian et al., 2022). VDAC governs mtDNA release in senescent cells (Lai et al., 2025), which critically depends on mtROS (Kim et al., 2025). Moreover, elevated mtDNA release in muscle fibers from progeroid mice has been demonstrated to rely on increased oligomerisation of VDAC1 (Li Y. et al., 2024). Acute severe hypoxia triggers sphingolipid metabolic disorders in mice, in which elevated C24-Ceramide (Cer) binds to VDAC1 to drive mtDNA leakage alongside inflammatory reactions and senescence-like phenotypic changes (Xu et al., 2026).

Oxidative stress and Ca^2+^ overload induce mPTP opening through the permeability transition pore complex (PTPC) embedded in the mitochondrial inner membrane (Bonora et al., 2022). In fact, studies have revealed that aging elicits an enhanced translocation of Ca^2+^ from the endoplasmic reticulum (ER) to mitochondria (Martin et al., 2023), and senescent cells adopt frequent mPTP opening to alleviate excessive intracellular Ca^2+^ accumulation (Protasoni et al., 2024). This indicates that aging-induced elevation of mitochondrial calcium load drives augmented mPTP opening, thereby markedly increasing inner mitochondrial membrane permeability and directly resulting in mitochondrial depolarization and boosted ROS production (Margand et al., 2025). Indeed, increased opening of the mPTP was observed in mitochondria harvested from aged rat cardiac tissue (Fernandez-Sanz et al., 2015), while aberrant mPTP opening is closely associated with neuronal loss in various neurodegenerative disorders that include AD and Parkinson’s disease (Muller et al., 2018). Intriguingly, aberrant mPTP opening is repressed under caloric restriction, which serves as a canonical longevity pathway (Amigo et al., 2017).

Overall, aging is accompanied by impaired mitochondrial clearance mechanisms and loss of mitochondrial integrity (Panel et al., 2018), which further facilitates the extracellular release of mtDAMPs, drives inflammatory responses and accelerates the aging process (Wei K. et al., 2024; Cai et al., 2025; Yu C. H. et al., 2020; Li Y. et al., 2024) (Figure 2A).

### 3.2 cGAS-STING signaling pathway

The cGAS-STING signaling pathway represents an immune program sensing leaked DNA, which exerts pivotal functions in antibacterial defense, inflammatory disorders, senescence, autoimmunity and tumorigenesis (Zhang and Zhang, 2025). Compared with nDNA, mtDNA acts as a potent activator of cGAS (Marchi et al., 2023; Kim et al., 2023). First, the structural features (lack of histone protection) and regional characteristics (proximity to the ETC) make mtDNA more vulnerable to oxidative damage, which disrupts mtDNA integrity and facilitates its release into the cytoplasm (Shimada et al., 2012; Kim et al., 2023). Furthermore, histones wrapping around nDNA occupy the DNA-binding sites of cGAS, which impedes cGAS dimerization and ultimately results in cGAS functional inactivation (Li et al., 2021; Zhao et al., 2020; Michalski et al., 2020). Besides, the cytoplasmic release of mtDNA takes place substantially earlier than that of nDNA (Lai et al., 2025).

Once mtDNA is released into the cytoplasm, the cGAS protein catalyzes cytoplasmic double-stranded DNA (dsDNA) molecules to produce the second messenger 2′3′-cGAMP (Zhang and Zhang, 2025; Ablasser et al., 2013; Li et al., 2013), which subsequently binds to STING anchored on the ER membrane. Consequently, activated STING translocates to the Golgi apparatus (Mukai et al., 2016), thereby recruiting tank-binding kinase 1 (TBK1) and IκB kinase (IKK). TBK1-dependent phosphorylation activates interferon regulatory factor 3 (IRF3), which dimerizes, translocates to the nucleus, and initiates transcription of type I interferons such as IFN-β1 (Dalskov et al., 2020; Zhao et al., 2019) (Figure 2B).

Indeed, numerous studies have verified that senescent cells exhibit increased mtDNA release (Lai et al., 2025; Li Y. et al., 2024). Mechanistically, apart from enhancing mitochondrial membrane permeability, senescence can also promote the extrusion of mtDNA by affecting mtDNA synthesis. For instance, senescence leads to disturbed nucleotide metabolism, causing elevated ribonucleotide misincorporation into mtDNA, which in turn promotes mtDNA extrusion (Bahat et al., 2025). Furthermore, senescence also elevates the levels of TBK1 and phosphorylated TBK1 (p-TBK1) (Gulen et al., 2023) as well as STING expression (Cai et al., 2024), which further facilitates the activation of the cGAS-STING signaling pathway. Notably, the aging-induced increase in STING promotes the dissociation of hexokinase 2 (HK2) from VDAC1, thereby facilitating the opening of the mPTP (Cai et al., 2024). All these age-dependent changes create a favorable environment for the activation of the cGAS-STING signaling pathway (Figure 3). However, in cardiomyocytes of APP/PS1 mice, cGAS-STING pathway activity was unexpectedly decreased, suggesting that these contradictory results may arise from differences in experimental conditions, pathological stages, and genetic backgrounds. In addition, depletion of Golgi-localized STING protein has been observed in brain tissues of AD patients, which may be attributable to chronic activation of cGAS-STING by accumulated dsDNA such as mtDNA, although whether this chronic activation pattern truly exists in the AD pathological environment remains to be confirmed (Quan et al., 2025).

**Figure 3:**
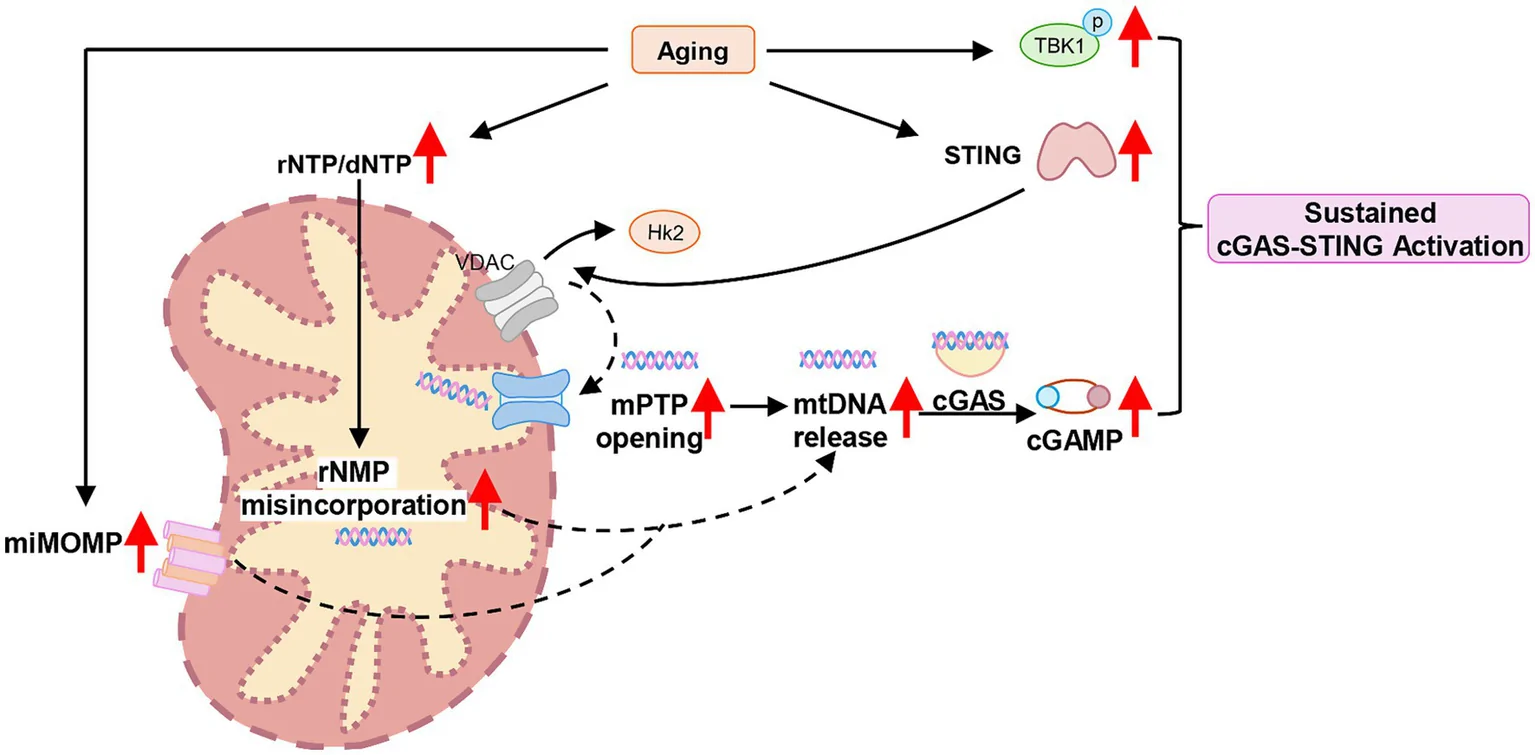
Age-associated alterations in multiple steps of the mitochondrial-related cGAS-STING pathway. Age-related increases in miMOMP opening, rNMP incorporation into mtDNA (which exacerbates mtDNA damage), mPTP opening, as well as elevated STING and TBK1 expression collectively promote cGAS-STING pathway activation. Upward arrows indicate increase. rNMP, ribonucleoside monophosphate; HK2, hexokinase 2; mPTP, mitochondrial permeability transition pore.

### 3.3 Inflammasome

A typical inflammasome consists of three core components, the first of which are sensor proteins belonging to the NOD-like receptor (NLR) family, which are primarily responsible for sensing danger signals and initiating complex assembly. The second component is the adaptor apoptosis-associated speck-like protein containing a CARD (ASC), which acts as a linker connecting the sensor segment to the effector segment. The last one is the effector pro-caspase-1, whose central function is to mediate proteolytic maturation of pro-IL-1β and pro-IL-18 so as to generate active inflammatory cytokines (Rathinam and Fitzgerald, 2016).

Recent studies have revealed that upon stimulation with NLRP3 inflammasome activators, HK2 dissociates from mitochondrial VDAC and translocates to the cytosol (Baik et al., 2023). This process triggers mitochondrial calcium overload through ER calcium efflux, driving VDAC oligomerisation and mtDNA release, whereby mtDNA, in turn, cooperates with oligomerized VDAC to recruit NLRP3 for inflammasome assembly (Baik et al., 2023). Furthermore, the activated NLRP3 inflammasome can in turn facilitate the release of oxidized mitochondrial DNA (ox-mtDNA) (Nakahira et al., 2011). Similar to NLRs, absent in melanoma 2 (AIM2) recognizes cytosolic dsDNA (Fernandes-Alnemri et al., 2009) as well as oxidative stress (Sridevi Gurubaran et al., 2022), whereas the AIM2 inflammasome preferentially senses unoxidized DNA (Zhong et al., 2018). Apart from mtDNA, ROS can likewise trigger inflammasome activation (Zhou et al., 2011; Casey et al., 2025). Moreover, the mitochondrially relocalized nuclear protein thioredoxin-interacting protein (TXNIP) and externalized cardiolipin can both form physical contacts with NLRP3 to drive inflammatory activation (Zhou et al., 2010; Saxena et al., 2010; Iyer et al., 2013). A recent study points out that cardiolipin can elicit conformational changes within NLRP3, alterations that further drive phase separation events that ultimately induce the activation of the NLRP3 inflammasome (Zou et al., 2025) (Figure 2B).

Interestingly, multiple studies have demonstrated a regulatory role for the cGAS-STING signaling pathway in inflammasome activity (Zhang W. et al., 2022; Liu J. et al., 2022; Zhong et al., 2020). For instance, sevoflurane-induced cytosolic release of mtDNA triggers inflammasome activation in a manner dependent on the cGAS-STING pathway (Yang N. S. et al., 2024). However, the precise mechanisms by which these two signaling pathways coordinate responses to mitochondrial dysfunction remain to be further elucidated.

### 3.4 NF-κB

A recent study has proposed that the integrity of the mitochondrial outer membrane directly governs NF-κB signaling (Vringer et al., 2024). The canonical NF-κB pathway relies on RelA-p50 heterodimer (Guo Q. et al., 2024). In resting cells, IκB sequesters RelA-p50 in cytoplasm. IKK-triggered IκB phosphorylation liberates the complex for nuclear translocation to regulate immunity, inflammation and cell-differentiation-related transcription (Yu H. et al., 2020).

NF-κB also functions downstream of the cGAS-STING signaling pathway to mediate immune responses triggered by mtDNA (Zhang B. C. et al., 2025). The kinase IKKε phosphorylates STING at the S358 residue, to which IRF3 is subsequently recruited and then binds. This event facilitates STING trafficking from the Golgi apparatus to endolysosomes and enables the recruitment of TNF-receptor-associated factor 6 (TRAF6), leading to subsequent NF-κB activation (Zhang B. C. et al., 2025). Notably, IFN is activated via Golgi STING, while NF-κB is driven by endolysosomal STING, and its nuclear translocation lags behind IRF3, revealing spatiotemporal specificity (Zhang B. C. et al., 2025) (Figure 2B).

mtRNA serves as an additional activator of the NF-κB signaling pathway and can be sensed by the three members of the RLR family. The retinoic acid-inducible gene I protein (RIG-I) recognizes short dsRNA, the melanoma differentiation-associated protein 5 (MDA5) detects long dsRNA, and laboratory of genetics and physiology 2 (LGP2) sequesters dsRNA. Upon binding to nucleic acids, RIG-I and MDA5 undergo conformational changes that expose their CARD domains, which then interact with mitochondrial antiviral signaling protein (MAVS) to trigger the downstream activation of IRF3 and NF-κB (Kalykaki et al., 2024; Hou et al., 2011; Seth et al., 2005). Furthermore, PKR and TLR3 can sense mtRNA and induce inflammatory gene expression via IRF3/7 and NF-κB, respectively (Chen et al., 2025b). Endosome-localized TLR8 recognizes single-stranded mtRNA. Exosome-packaged mitochondrial double-stranded RNA (mt-dsRNA) from neighboring cells enters recipient cells via endocytosis and is sensed by intracellular TLR3 to trigger inflammation. The mt-dsRNA drives PKR dimerization and autophosphorylation to activate the NF-κB pathway (Chen et al., 2025b) (Figure 2B).

Recent studies reveal that mitochondrial ubiquitination regulates the NF-κB signaling pathway. Since mitochondrial ubiquitination promotes NF-κB nuclear import and PINK1 antagonistically inhibits deubiquitination, mitochondria serve as an amplification platform for NF-κB signals (Wu et al., 2022). Further studies demonstrate that oxidative damage triggers Parkin-dependent mitochondrial ubiquitination, which does not serve as an autophagy-inducing “eat-me” signal but recruits NEMO to depolarized mitochondria to launch NF-κB signaling via phosphorylated IKKβ (Harding et al., 2023) (Figure 2C). These findings reveal that distinct subsets of damaged mitochondria drive divergent downstream signaling pathways.

### 3.5 Reciprocal vicious cycles of aging hallmarks

Senescence constitutes an evolutionarily conserved protective mechanism that eliminates senescent cells through innate and adaptive immunity to restrain the proliferation of potentially harmful cells. In fact, senescent cells deploy multiple immune evasion mechanisms, and age-related deterioration of the immune system further favors accumulation of senescent cells (Majewska and Krizhanovsky, 2025), which in turn drives the buildup of dysfunctional mitochondria. We have elaborated above how aging-induced mitochondrial dysfunction drives the activation of chronic inflammation. In turn, this activated inflammatory signaling exerts reciprocal effects on mitochondria, which exacerbates the accumulation of mitochondrial defects. For example, activation of the AIM2 and NLRP3 inflammasomes drives excessive mitochondrial ROS generation, inner mitochondrial membrane depolarization, increased permeability and mitochondrial network fragmentation (Yu et al., 2014). Moreover, inhibition of mitophagy by caspase-1 further increases the abundance of dysfunctional mitochondria (Yu et al., 2014). Baechle et al. (2023) place chronic inflammation at the center of the aging process, positing that it interacts reciprocally with the other 11 hallmarks of aging. Indeed, it remains challenging to disentangle the causal relationships between mitochondrial dysfunction, chronic inflammation and other hallmarks of aging, as these factors act as mutually reinforcing triggers that accelerate organismal deterioration.

## 4 Mitochondrial-inflammatory dyshomeostasis in AD

Given that mitochondrial dysfunction and chronic inflammation, which are inevitable hallmarks of aging, jointly contribute to the pathological progression of AD, it can be inferred that aging drives the onset of AD, at least in part, through this mitochondria-inflammation pathological axis.

Reduced mitochondrial abundance observed in AD patients leads to inadequate ATP synthesis, eliciting a GSH crisis that ultimately drives neuronal ferroptotic injury (Alves et al., 2025). Recent studies have fused mtDNA-depleted SH-SY5Y cells with platelets isolated from healthy individuals, patients with cognitive impairment, and patients with AD, respectively. The total tau protein and phosphorylated tau (p-tau) levels were markedly elevated in both the AD group and the cognitive impairment group (Kemna et al., 2025). Taken together, age-related mitochondrial deficiency, such as impaired ATP production and alterations in mtDNA, may constitute critical inducers of AD. Indeed, as mtDNA release increases with aging, it activates the cGAS-STING pathway that produces senescence-associated inflammatory signals, which in turn results in physical and cognitive decline (Gulen et al., 2023). Moreover, increased cGAS-STING activity is observed in AD mice (Hou et al., 2021). Fragmented Ox-mtDNA, which is released into the cytosol as Z-DNA, is recognized by Z-DNA-binding protein 1 (ZBP1) and activates downstream immune responses via receptor-interacting protein kinase 1 (RIPK1), which in turn gives rise to AD-associated microglial neuroinflammation (Song et al., 2025). Strikingly, recent studies highlight that inhibiting inflammatory cascades represents a core therapeutic strategy for AD (Feng et al., 2026).

mtDAMPs that accumulate over time sustain persistent activation of inflammatory signaling pathways, which triggers robust secretion of pro-inflammatory cytokines that continuously compromise neuronal and synaptic integrity. This cumulative neuroinflammatory signal may contribute to initiating and propagating the full pathological cascade of AD, and also serves as the critical pathological link that bridges aging-related mitochondrial lesions to AD-driven neurodegeneration.

### 4.1 Inflammation and blood–brain barrier (BBB) dysfunction

The BBB, which constitutes a specialized interface between the brain and blood and is composed of endothelial cells, pericytes and astrocytes, serves as a vital structure that maintains normal brain physiological functions, as it prevents peripheral toxins and immune cells from infiltrating brain tissues through physical segregation (Yoon et al., 2025). However, the BBB, whose barrier function becomes disrupted during aging and neurodegenerative diseases, may even give rise to cerebral hemorrhage in mice (Shi et al., 2025). Since increased BBB permeability emerges prior to the deposition of Aβ plaques and neurofibrillary tangles, this pathological feature implies that the BBB could serve as a biomarker for early diagnosis of AD (Lee and Funk, 2023). Activation of inflammatory signaling cascades constitutes one major cause of BBB dysfunction, which can be exemplified in cases of radiation-mediated brain endothelial damage where cytoplasmic mtDNA release activates the cGAS-STING axis that initiates type I interferon responses and disrupts intact BBB function (Wang P. et al., 2025). Peripheral recurring inflammation, which triggers activation of the NLRP3-GSDMD signaling axis within microglial cells, promotes GDF-15 secretion through an IL-1β-independent pathway that drives C-X-C motif chemokine ligand (CXCL) chemokine expression to recruit neutrophils releasing matrix metalloproteinases (MMPs) that eventually destroy the integrity of the BBB (Yoon et al., 2025). Interestingly, CXCL and MMPs, which belong to SASP factors, show elevated secretion levels with aging (Li et al., 2023). It is worth noting that during healthy aging, the BBB undergoes a loss of structural integrity that arises from elevated basal neuroinflammation, which is manifested by the degeneration of pericytes and astrocytes, disruption of tight junctions and downregulation of transporters that collectively drive the imbalance of the central nervous system microenvironment (Lee and Funk, 2023). These observations may suggest that SASP acts as a critical factor that triggers BBB damage (Figure 4A).

**Figure 4:**
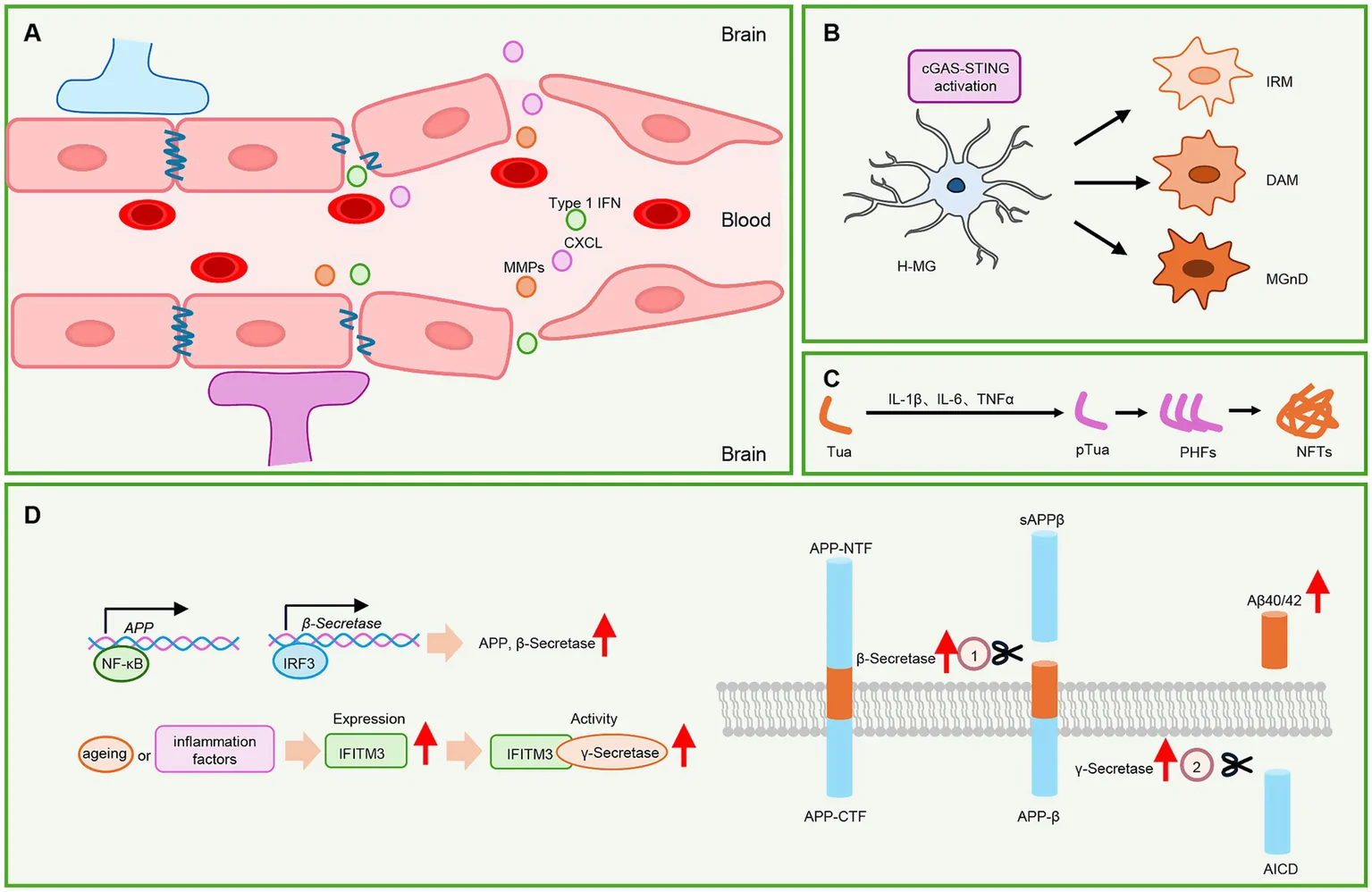
Schematic illustration of mechanisms whereby inflammatory signals trigger AD pathological progression. **(A)** Inflammatory signals impair the integrity of the blood–brain barrier. **(B)** cGAS-STING-driven microglial phenotypic transition toward IRM, DAM, and MGnD subsets. **(C)** Inflammatory signalling molecules trigger tau phosphorylation and consequently facilitate its pathological aggregation. **(D)** Inflammatory signalling pathways promote the transcription and cleavage of APP, leading to Aβ production. Upward arrows indicate increase. CXCL, C-X-C motif chemokine ligand; MMPs, matrix metalloproteinases; H-MG, homeostatic microglia; IRM, interferon-responsive microglia; DAM, disease-associated microglia; MGnD, neurodegenerative microglia; PHFs, paired helical filaments; NFTs, neurofibrillary tangles; APP, amyloid precursor protein; IFITM3, interferon-induced transmembrane protein 3; APP-NTF, APP N-terminal fragment; APP-CTF, APP C-terminal fragment; AICD, APP intracellular domain.

The structural damage that occurs to the BBB during aging means that low-grade chronic inflammation and other toxic substances derived from peripheral tissues are no longer restricted to the peripheral tissue microenvironment, which permits them to continuously infiltrate the central nervous system and disrupt the inherent steady immune homeostasis in the brain that gradually evolves into a neuroinflammatory state marked by excessive microglial activation as its core feature.

### 4.2 Neuroinflammation and microglial

As resident immune cells of the central nervous system, microglia mediate Aβ clearance under resting conditions. A study of 84 elderly donors (mean age at death 88 years, covering a continuous pathological spectrum ranging from no AD pathology to severe AD pathology) revealed microglial inflammatory activation at the early stage of AD, accompanied by upregulation of multiple inflammatory signaling pathways (Gabitto et al., 2024). According to the neuroinflammatory hypothesis, chronic inflammation triggered by aging serves as priming stimulation that primes microglia into a “primed” phenotype, and subsequent pathological insults induce persistent harmful microglial activation (Leng and Edison, 2021).

The traditional M1-M2 dichotomy simply classifies microglia into the M1 phenotype (which secretes pro-inflammatory cytokines and exacerbates neuronal death) and the M2 phenotype (which suppresses inflammation and is generally repressed in AD) (Yang et al., 2025). Nevertheless, such simplistic classification fails to truly capture the complex continuum of microglia *in vivo*. Single-nucleus RNA sequencing analyses have further subdivided them into homeostatic microglia (H-MG), interferon-responsive microglia (IRM), disease-associated microglia (DAM), and neurodegenerative microglia (MGnD) (Quan et al., 2025). H-MG represents a population of ramified resting microglia with immune surveillance and homeostatic maintenance functions. Notably, cGAS activation alone is sufficient to drive the transformation of H-MG into IRM, DAM, and MGnD (Figure 4B) (Quan et al., 2025; Gulen et al., 2023). IRM exhibits cGAS-dependent upregulation of type I interferon-stimulated genes, including *Ifits*, *Oasl1*, *Oasl2*, *Isg15*, and *Stat2*. DAM is a microglial subset that recognizes neurodegeneration-associated molecular patterns (NAMPs). A prevailing view holds that DAM function is time-dependent, in that it mediates protective immune responses in early-stage AD, while persistent plaque-driven inflammasome activation in late AD induces DAM dysfunction to accelerate disease progression (Deczkowska et al., 2018). cGAS activation drives the conversion of low-activation DAM-1 to hyperactivated DAM-2, with concomitant upregulation of lysosomal and lipid metabolic genes (*Ctsb*, *Ctsd*, *Ctsz*, *Cd9*, *Cd63*, *Apoe*) (Quan et al., 2025, Gulen et al., 2023). MGnD represents a microglial subset that highly expresses neurodegeneration signature genes including *Apbb2*, *Olfr111*, and *Xylt1* (Quan et al., 2025, Gulen et al., 2023). However, beyond the cGAS-STING signaling pathway, whether other inflammatory signaling pathways are directly involved in the regulation of microglial subtypes remains to be further investigated.

### 4.3 Neuroinflammation and tau hyperphosphorylation

Tau protein, encoded by the MAPT gene, localizes to neuronal axons under physiological conditions, where it facilitates microtubule assembly and stability and participates in axonal transport as well as neuronal outgrowth. Post-translational modifications of tau protein, particularly phosphorylation, drive tau aggregation into insoluble neurofibrillary tangles (NFTs) and soluble oligomers (Chen and Yu, 2023), whose pathological aggregation shows a strong correlation with cognitive impairment and AD progression. Intriguingly, recent studies further recognize the D-peptide, which possesses potent activity to disassemble AD-related tau aggregates, as a promising therapeutic candidate (Hou et al., 2025). Furthermore, the plasma %p-tau217 clock model serves as a predictive tool to estimate the time required for cognitively normal individuals to progress to symptomatic AD (Petersen et al., 2026). More importantly, experimental results demonstrate that inflammatory cytokines induce bead-like tau aggregates within axons and dendrites, and the abundance of such beads rises in the hippocampus and cortex of mice with advancing age (Tseng et al., 2017). Upon recognizing pathological stimuli, microglia initiate immune responses, through which inflammatory cytokines further modulate the phosphorylation level of tau protein (Figure 4C) (Chen and Yu, 2023). For instance, after acting on neurons, cytokines such as IL-1β directly modulate the phosphorylation levels of tau at multiple sites by activating p38α mitogen-activated protein kinase and glycogen synthase kinase-3β (Li et al., 2003; Maphis et al., 2016; Peng et al., 2023; Chakraborty et al., 2024). In addition, pro-inflammatory cytokines including IL-6 and TNF-α exert their regulatory effects via the cyclin-dependent kinase 5 kinase-dependent pathway (Zhang L. et al., 2022; Quintanilla et al., 2004). Primary neuronal cells treated directly with IL-6 exhibit excessive phosphorylation of tau at the Ser396 and Ser404 residues (Weston et al., 2021). More importantly, while pro-inflammatory cytokines induce excessive phosphorylation and aggregation of tau, tau protein itself can serve as an activator of NLRP3, thereby forming a positive feedback loop that ultimately exacerbates disease progression (Ising et al., 2019). Astrocytes stimulated by pro-inflammatory cytokines TNF-α and IL-1β upregulate glypican-4 (GPC-4) through the NF-κB signaling pathway, whose secreted GPC-4 protein binds to apolipoprotein E4 to promote trafficking of low-density lipoprotein receptor-related protein 1 receptors on neuronal surfaces, a process that further boosts neuronal tau uptake and phosphorylation (Saroja et al., 2022). These findings imply that aging-triggered neuroinflammation may serve as a critical driver of pathological tau aggregation.

### 4.4 Neuroinflammation and aβ accumulation

Neuroinflammation can modulate Aβ synthesis, proteolytic cleavage and degradation, thus engaging in the pathological progression of Aβ accumulation in AD. Amyloid precursor protein (APP) undergoes sequential proteolytic cleavage by β-secretase (BACE1) followed by γ-secretase to generate highly pathogenic Aβ40 and Aβ42 peptides. Notably, the promoter regions of APP and BACE1 (the rate-limiting enzyme governing amyloidogenic cleavage) contain binding motifs for inflammation-associated transcription factors, which accounts for their elevated transcription upon inflammatory stimulation (Heneka et al., 2025). Furthermore, both aging and inflammatory stimuli trigger upregulation of interferon-induced transmembrane protein 3 (IFITM3), and the enhanced interaction between IFITM3 and γ-secretase potentiates its proteolytic cleavage activity, ultimately leading to elevated Aβ levels (Hur et al., 2020). The NF-κB pathway, which is activated by excessive mitochondrial ROS production resulting from PINK1 deficiency, enhances BACE1 and γ-secretase activity, thereby promoting substantial Aβ accumulation (Du et al., 2025). Meanwhile, diminished protein degradation contributes to protein aggregation (Figure 4D). For instance, evidence from AD mouse models directly confirms that NLRP3 inflammasome activation markedly suppresses cellular Aβ clearance (Tejera et al., 2019).

## 5 Mitochondria-targeted therapeutic strategies for AD

In fact, beyond modulating AD pathogenesis through inflammatory responses, mitochondrial dysfunction itself constitutes a critical driver of AD progression. First, mitochondrial dysfunction that precedes Aβ aggregation and tau pathological alterations suggests that it constitutes an early pathological event of AD, and such mitochondrial defects can not only promote Aβ generation and cleavage but also aggravate tau hyperphosphorylation and subsequent tangle deposition (D'Alessandro et al., 2025). Consequently, mitochondrial-centered strategies focused on inhibiting inflammation may solve both mitochondrial and chronic inflammatory problems, thus holding great promise as preventive and forward-looking therapeutic tools for AD.

### 5.1 Mechanisms and limitations of mitochondria-targeted drugs

While these mechanistic observations offer a potential rationale for mitochondria-targeted AD interventions, substantial translational challenges remain when translating preclinical findings to human clinical application. Notably, phase II candidates, such as rapamycin, astragalus, resveratrol, and curcumin, account for the largest proportion (approximately 33%) among all clinically tested agents (Zhou et al., 2026). While these data suggest that such agents show promise in early-stage studies, only a small fraction proceed to later-phase trials, highlighting translational bottlenecks for this therapeutic class.

Rapamycin, a classic mTOR inhibitor, suppresses protein translation and promotes autophagy, and thus represents a classic geroprotective agent (Selvarani et al., 2021). From a mitochondrial perspective, the pathways through which rapamycin acts involve activating mitophagy and reducing ROS. In addition, its regulatory effects on Aβ and Tau are also important mechanisms underlying its therapeutic efficacy (Hou et al., 2023). However, the practical application of rapamycin is subject to numerous limitations. First, model differences, routes of administration, dosage, timing of administration, and targeted delivery vehicles can all influence the experimental outcomes. Second, studies have shown that early intervention is associated with better efficacy, whereas in advanced AD the benefits are limited or even detrimental. Third, chronic microglial overactivation that promotes inflammation and damages neurons. Finally, long-term use of the drug is associated with side effects, which manifest as glucose intolerance, diabetes, immunosuppression, and others (Hou et al., 2023).

A previous phase I clinical trial administered rapamycin at 1 mg daily for 8 weeks. However, the drug was not detected in the cerebrospinal fluid of the participants, and the relevant pathological markers showed no improvement (Gonzales et al., 2025). In another phase IIa clinical study, although intermittent dosing was adopted, current detection methods still cannot reliably assess cerebrospinal fluid (CSF) drug concentrations (with free drug concentrations in CSF below the lower limit of detection), leaving central drug exposure in the human brain unclear (Wallgren et al., 2026). Notably, an ongoing phase II clinical trial (NCT04629495) is further evaluating the safety, tolerability, and feasibility of rapamycin^1^. Therefore, in the future, while improving detection methods to determine whether rapamycin can cross the BBB, developing effective and safe brain-targeted delivery systems remains an urgent challenge to be overcome.

Astragalus, a natural medicinal product, contains various active components, including saponins, flavonoids, and polysaccharides (Dong et al., 2022). The total extract of astragalus can improve learning and memory abilities in AD mice (Li et al., 2022). Astragaloside IV (AS-IV), a core bioactive component of astragalus, inhibits the opening of the mPTP, reduces ROS, decreases cytochrome c release, protects mitochondria, and attenuates inflammatory activation (Li et al., 2022). However, astragalus suffers from significant metabolic degradation after oral administration and insufficient BBB penetration, which constitute major translational challenges (Li et al., 2022). A phase I clinical trial of AS-IV injection has provided the basis for determining its therapeutic dose (Zou et al., 2016). Currently, two phase II clinical trials (NCT05578443 and NCT06694597) evaluating the safety and therapeutic efficacy of astragalus for the treatment of AD are ongoing (see text footnote 1).

As an AD therapeutic candidate, resveratrol functions through the sirtuin 1-adenosine monophosphate-activated protein kinase bidirectional regulatory axis to activate mitophagy, facilitate mitochondrial biogenesis, block mPTP opening, stimulate antioxidant pathways and relieve neuroinflammation (Al-Dhahi et al., 2026). Preclinical data indicate that resveratrol can extend the lifespan of nematodes and alleviate AD pathology in mice (Al-Dhahi et al., 2026). However, actual clinical data show that resveratrol has poor bioavailability and limited efficacy in humans (Zhou et al., 2026; Puranik et al., 2025).

Curcumin is a natural polyphenol found in turmeric. In AD models, curcumin can alleviate AD symptoms by inhibiting Aβ/Tau aggregation, counteracting oxidative stress, exerting anti-inflammatory effects, chelating metal ions, modulating epigenetic regulation, and regulating neurotransmitters (Abdul-Rahman et al., 2024). However, clinical trial results remain highly inconsistent, and there is no conclusive evidence that curcumin is effective in patients with AD, largely because its oral bioavailability is extremely low and its ability to cross the BBB is poor (Abdul-Rahman et al., 2024).

According to statistics, 58.9% of mitochondria-targeted Phase II clinical candidates are based on anti-oxidative stress, whereas relatively few agents target mitochondrial dynamics and mitophagy (Zhou et al., 2026). These figures reflect an unbalanced development of therapeutic targets for mitochondrial-related interventions. In addition to these drugs that are currently in clinical trials, there are still many mitochondria-targeted candidates that remain in preclinical development and that, while they may have therapeutic potential for AD. Therefore, based on the mechanism by which mitochondrial dysfunction triggers inflammation, we focus on candidates that satisfy at least one of the following criteria, namely lowering overall mtDAMPs burden independent of mitochondrial removal, enhancing the clearance of impaired mitochondria, and inhibiting the opening of release channels (Table 1).

**Table 1:** Preclinical candidates targeting the mitochondrial-inflammation axis with potential for AD therapy.

Drugs	Findings	Core limitations	References
MitoQ and SkQ1	MitoQ and SkQ1 accumulate in the mitochondrial matrix by virtue of the negative transmembrane potential of mitochondria, scavenging mtROS.	MitoQ and SkQ1 have poor BBB penetration, carry a risk of off-target effects, and have a narrow therapeutic window (high doses can exert pro-oxidant effects).	Song (2026)
Urolithin A (UA)	In multiple AD mouse models, UA ameliorates cognitive decline by restoring mitophagy and lysosomal function.	Although UA can enter the brain, its effective brain concentration and pharmacokinetics remain unclear. Its efficacy varies widely due to gut microbiota-dependent metabolism, and excessive mitophagy may cause non-selective autophagy in peripheral tissues (e.g., the gut), raising the risk of off-target effects.	Hou et al. (2024) and Bisht et al. (2026)
Kaempferol and rhapontigenin	By activating PINK1-Parkin-dependent mitophagy, kaempferol and rhapontigenin ameliorate memory function in AD models of nematodes and mice.	Kaempferol can cross the BBB, but it is uncertain whether rhapontigenin can. Excessive activation of mitophagy may be detrimental.	Xie et al. (2022)
Honokiol	Honokiol eliminates damaged mitochondria via mitophagy, reduces oxidative stress, attenuates Aβ aggregation, alleviates hippocampal neuronal damage, and improves cognitive performance in APP/PS1 mice.	Although honokiol has been shown to cross the BBB in mice, there is currently no evidence that it can cross the human BBB.	Li H. et al. (2024) and Lin et al. (2012)
Aloe-Emodin (AE)	Via the activation of neuronal mitophagy, AE attenuates hippocampal neuronal damage and improves cognitive impairment in APP/PS1 AD mice.	AE is relatively sensitive to some targets, and the complete dose-effect relationship for each target remains unclear. The experiment only explored 6-month-old AD mice, and the therapeutic effect of AE in the late stage of AD remains unknown.	Wang Y. et al. (2025)
Cyclosporine A	Cyclosporine A diminishes cleaved-tau induced by caspase-3 and blocks mPTP pore opening, thereby reversing age-related cognitive impairment as well as mitochondrial dysfunction.	Cyclosporine A exerts potent immunosuppressive effects, with poor central nervous system specificity and limited BBB penetration.	Tapia-Monsalves et al. (2023), D'Alessandro et al. (2025), and Samanta et al. (2024)
Ebselen	As a BBB-permeable covalent inhibitor of CypD, ebselen restores mitochondrial membrane function and lowers ROS via inhibition of the CypD-mPTP pathway, and improves cognitive ability in two AD transgenic mouse models including mAPP and 5 × FAD.	Ebselen is a compound that covalently modifies cysteine residues and can target cysteines in multiple proteins, giving rise to off-target risks.	Samanta et al. (2024)
VBIT-4	VBIT-4 inhibit the oligomerization of outer mitochondrial membrane VDAC1 to block the release of mtDNA, ROS and pro-apoptotic factors, and alleviate tissue pathological injuries in AD.	Target selectivity remains to be further confirmed, with potential off-target risks.	Shteinfer-Kuzmine et al. (2026)
Mdivi-1	Mdivi-1 can inhibit mitochondrial fission, reducing the accumulation of fragmented mitochondria, and decreasing Bax/Bak-mediated MOMP in a DRP1-dependent manner. Besides, it suppresses ROS by inhibiting complex I reverse electron transfer.	Mdivi-1 faces three key barriers to clinical translation. First, its complex mechanism makes it unclear whether *in vivo* efficacy is driven mainly by DRP1 or complex I inhibition. Second, its toxicity is cell-type-dependent, as it protects neurons but damages glial cells *in vitro*. Third, its therapeutic window is narrow because DRP1-inhibitory concentrations also inhibit complex I.	Liu X. et al. (2022) and Zhou et al. (2026).
SS-31	SS-31, through binding to cardiolipin, maintains the structural integrity of mitochondrial inner membrane cristae and stabilizes respiratory chain supercomplexes, thereby reducing electron leakage and ROS production. Moreover, SS-31 inhibits the opening of the mPTP, an effect that alleviates calcium overload and mitochondrial swelling	The main factors limiting the translation of SS-31 include limited BBB penetration, extremely poor oral bioavailability (with reliance mainly on injectable administration), and a short plasma half-life requiring frequent dosing.	Reddy et al. (2017)

In addition, mitochondrial transplantation (Zhou et al., 2026) and endogenous peptides [such as mitochondrial-derived micropeptides Humanin (Alqahtani et al., 2025) and the endogenous neuropeptide PACAP (Basri et al., 2026)] represent promising therapeutic strategies for AD. Mitochondrial transplantation can directly re-establish mitochondrial homeostasis and rescue impaired energy metabolism, whereas endogenous peptides have favorable biocompatibility and low toxicity and can alleviate oxidative stress and neuroinflammation. Moreover, tirzepatide, a dual GLP-1/GIP receptor agonist, holds therapeutic potential for AD, since it can cross the BBB to activate antioxidant signaling pathways and suppress inflammatory signals (Alshehri et al., 2025). Nevertheless, like mitochondrial transplantation and endogenous peptides, all these approaches rely largely on preclinical findings, with large-scale human clinical data still absent. Notably, several agents that dampen inflammatory responses by limiting the release of mtDAMPs still exhibit promising therapeutic potential, although they have not been further validated in AD animal models, as exemplified by deoxyribonucleosides (Bahat et al., 2025) and zinc (Jin et al., 2026).

### 5.2 Origins of translational barriers in mitochondria-targeted AD therapeutics

Although many mitochondria-targeted therapeutic candidates for AD have shown promise at the research and development stage, they remain a long way from routine clinical implementation. In fact, the slow clinical progress of mitochondria-targeted drugs is mainly associated with AD characteristics and intrinsic drug deficiencies.

From the perspective of disease characteristics, AD has a long asymptomatic latent period in its early stage. Mitochondrial dysfunction occurs prior to Aβ- and tau-mediated AD pathological alterations (D'Alessandro et al., 2025). By the time patients present with memory decline, substantial neuronal loss has often already taken place. Mitochondria-targeted interventions administered at this stage can hardly reverse established neuronal damage and only partially slow disease progression, which makes it difficult to observe significant improvements in cognitive function. Furthermore, AD is driven by multiple interwoven pathological pathways, in which pathogenic factors including Aβ deposition, tau hyperphosphorylation, neuroinflammation, calcium homeostasis disturbance and mitochondrial dysfunction interact and modulate one another. Accordingly, single-target intervention merely targeting mitochondria is insufficient to counteract the overall pathological damage. Moreover, AD exhibits substantial patient-to-patient heterogeneity. Differences in age at onset, Aβ/tau pathological burden, degree of mitochondrial damage and comorbidities can lead to variable therapeutic responses among patients under standardized dosing regimens. Notably, AD is modulated by multiple factors, including sex differences (Lopez-Lee et al., 2024), APOE genotype (Zhang R. et al., 2022), TREM2(Zhang L. et al., 2025), peripheral immune (Zhang et al., 2024b; Fawzy, 2026), vascular (Abbas et al., 2026; Li J. et al., 2025), lifestyle/environmental factors (Li J. et al., 2025), and microbiota-gut-brain axis (Fawzy and Fathy, 2026). These factors interact with one another and may, directly or indirectly, influence mitochondrial function and inflammatory responses, thereby affecting the therapeutic efficacy of mitochondria-targeted drugs. For example, sex differences are partly attributable to the decline in estrogen after menopause, which weakens mitochondrial protection, leading to impaired brain energy metabolism, oxidative stress, and reduced Aβ/tau clearance, thereby promoting AD onset and progression (Lopez-Lee et al., 2024). They should be considered important limitations and future research directions.

Besides, numerous intrinsic drawbacks also reside in the drugs themselves. The primary concern is the insufficient BBB penetration of drugs, as exemplified by curcumin, a natural product whose favorable preclinical activity is compromised by its poor BBB permeability, which leads to limited drug exposure in brain tissues and thus constrains its clinical efficacy. Secondly, insufficient drug specificity can trigger off-target toxicity. Mitochondria-targeted agents for neurological diseases need to specifically act on neuronal mitochondria, yet administration routes such as injection and oral intake inevitably exert effects on mitochondria in peripheral tissues, thereby increasing the risk of side effects. Thirdly, poor drug stability renders the agents susceptible to peripheral metabolic clearance *in vivo*, which necessitates frequent administration. Lastly, there exists a preclinical-to-clinical translational gap. It is noteworthy that many proposed pathogenic mechanisms of AD are derived from cellular or animal AD models, yet these models cannot fully recapitulate human AD pathology. Animal models differ from humans in pathogenesis, physiological structure and genetic background. Meanwhile, they exhibit relatively simple pathological features, whereas human patients suffer from complex pathological superimposition. This accounts for why some candidate drugs with favorable efficacy in animal studies show mediocre performance in human trials.

### 5.3 Future perspectives

Given the disease characteristics of AD and multiple limitations of existing mitochondria-targeted drugs, to advance the development of mitochondrial-targeted therapeutic strategies for AD, future research should at least focus on optimization from the following six aspects.

First, explore broad-spectrum clinical biomarkers for early-stage AD, especially mitochondria-related biomarkers, so that mitochondria-targeted interventions can be initiated at the early disease stage to achieve the preventive treatment of AD. Second, carry out comorbidity-related research. Preclinical studies should adopt comorbidity models to evaluate drug efficacy and safety. In addition, clinical trials should enroll subjects with underlying comorbidities to systematically assess drug–drug interactions. Third, promote synergistic combination therapeutic strategies that integrate mitochondrial-targeted drugs with Aβ/Tau-targeted approaches. Fourth, improve the BBB permeability of drugs through molecular structural modification, carrier-mediated delivery systems such as liposomes and nanocarriers, as well as cell-based delivery systems. Fifth, develop AAV-mediated gene therapy strategies. For instance, AAV9-DJ-1 can enhance antioxidant capacity and mitophagy in APP/PS1 model mice, thereby effectively improving their cognitive function (McGill Percy et al., 2025). Sixth, promote large-sample and multicenter clinical trials to comprehensively evaluate the clinical safety and efficacy of drugs.

The synergistic implementation of the above multi-dimensional strategies is expected to break the clinical transformation bottleneck of mitochondrial-targeted drugs for AD and provide novel insights and directions for the precise intervention of this disease.

## Footnotes

1
www.clinicaltrials.gov
